# Factors of Airline Selection and Reflight Intention During the Pandemic/Case of Serbian Airlines Users

**DOI:** 10.3389/fpsyg.2022.915321

**Published:** 2022-07-04

**Authors:** Milica Aleksić, Jovanka Popov Raljić, Tamara Gajić, Ivana Blešić, Miloš Dragosavac, Mirjana Penić, Jovan Bugarčić

**Affiliations:** ^1^Faculty of Tourism and Hotel Management (FTH), University of Business Studies, Banja Luka, Bosnia and Herzegovina; ^2^Faculty of Tourism and Hotel Management, Singidunum University, Belgrade, Serbia; ^3^Geographical Institute “Jovan Cvijić” SASA, Belgrade, Serbia; ^4^Institute of Sports, Tourism and Service, South Ural State University, Chelyabinsk, Russia; ^5^Faculty of Hotel Management and Tourism, University of Kragujevac, Vrnjačka Banja, Serbia; ^6^Department of Geography, Tourism and Hotel Management, The Faculty of Science, University of Novi Sad, Novi Sad, Serbia; ^7^Modern Business School, Belgrade, Serbia

**Keywords:** airline selection, re-flight intention, COVID-19, airlines users, food quality and safety

## Abstract

The global pandemic coronavirus disease 2019 (COVID-19) has caused significant economic changes for all segments of the economy. Travel restrictions have landed several commercial airlines and significantly reduced their revenues. Safety measures are strict and very demanded, especially when it comes to food drinks and beverages served during flights. This article aims to discover the predictors that influenced the intention of the airline’s passengers to travel long-distance flights in unusual conditions of the COVID-19 pandemic and differs from current studies on airline selection and passenger loyalty because it includes changes in the behavior of employees who regularly fly medium- and long-distance flights. Requirements for passenger’s airline selection have been changed, which is why this study aimed to determine which factors influence the selection during reopening after lockdown. Determinants of food quality and safety during flights are a long-term challenge and could affect passengers’ choice of the airline they want to fly. This study was conducted during the reopening period of airlines, during the COVID-19 pandemic, on a sample of 369 Serbian passengers and employees on medium- and long-distance flights, in the period from November 20, 2020 to January 15, 2021. Regression analysis concluded that certain predictors such as food service quality and safety significantly affect the attitude, subjective norms, and perceived behavioral control (PBC) of passengers and trigger the intention that affects behaviors in the choice of the airline during the COVID-19 pandemic, especially when it comes to the flights with medium and long durations. To better interpret the effects, a path analysis was performed in the SPSS Analysis of Moment Structures (AMOS) software, version 26.00 with the aim to examine the importance and significance of causal relationships between groups of variables. The results confirmed the theory of planned behavior; that intentions are a significant mediator between the mentioned independent variables (attitudes about quality and safety of food, drinks and beverages, subjective norms, and perceived behavior control) and passenger behavior when rechoosing the same airline.

## Introduction

Coronavirus disease 2019 (COVID-19) has caused enormous damage to airlines globally. According to the International Civil Aviation Organization (ICAO, 2022), the extent of the effects of the tourism crisis is partly known and the severity varies at the level of different countries. Social distancing and closure have significantly affected the transport sector, especially the airline industry, which remains very fragile and one of the biggest victims of the COVID-19 pandemic. The International Civil Aviation Organization (2022) estimated actual results for 2020 and 2021, compared to 2019 levels, which showed a total reduction of 40–50% of airline supply. Fear appeal and social media fake news during COVID-19 have had a strong positive impact on impulse buying as mediating factors (Ahmed et al., 2020). When it comes to the decision of airline selection and reflight intention during the COVID-19 pandemic, the mentioned mediators had the opposite effect and they had a strong negative impact on the behavior of passengers. During the critical period of the COVID-19 pandemic, people were most afraid of infection during travel and lack of funds and job loss (Gajić et al., 2021a). Damage and uncertainty caused by the COVID-19 pandemic may have already permanently affected changes in passenger behavior (Song and Choi, 2020; Priem, 2021; Watson and Popescu, 2021), which is why it is necessary to pay attention to changes in behavior, especially when it comes to sensitive services such as food, drinks, and beverage services on long-distance flights.

This study has been one of the pioneering studies that investigate the factors affecting the airline travel intention of Serbian passengers during the COVID-19 disease period and aim to provide a guide to the airline managers for future projections in the passenger traffic by discovering the airline passengers’ behavior in the COVID-19 era, based on the Theory of Planned Behavior (TPB). The main objective of this study is to point out to the airlines’ management how certain predictors with more or less significance affect the attitude, subjective norms, and perceived behavior control (PBC) of Serbian passengers and trigger an intention that then trigger refight behavior during a pandemic, especially when it comes to medium- and long-duration flights. Predictors of intention are important in predicting the planned behaviors of passengers whose reselection of the airline has a positive impact on competitiveness, business improvement, and financial benefits. In the second part of this study, hypotheses based on current literature will be set. After reviewing the literature and methodology, the results are reviewed and discussed.

## Literature Review

To run a sustainable business in today’s tough global competitive climate and create a long-term base of loyal customers, airlines should be able to continuously provide quality food, drinks, and beverages service to their customers. Satisfaction of service users and employees are key determinants of loyalty, necessary for the existence and sustainability of business (Özkul et al., 2020). Providing high-quality services to passengers and tourists can be crucial for the competitiveness, profitability increasing, and long-term growth of airlines in a highly competitive environment, which is why they must meet the expectations of their passengers to maintain business (Arasli et al., 2020; Gajić et al., 2021b; Bakır et al., 2022). The quality of service in air transport is summed up by the results of several studies and presented in a study by Fu (2019). The study says that in forming the experience of service provided by airlines, the following factors play an extremely important role: accuracy of service, flight check-in, cabin comfort, after-sales services, service of food, drinks, and beverages, and programs for frequent flyers [frequent flyer programs (FFPs)].

Thamagasorn and Pharino (2019) stated that there is a lack of adequate study on this topic and conclusions, which would greatly improve the concept of total quality management (TQM) in the aviation industry.

**FIGURE 1:**
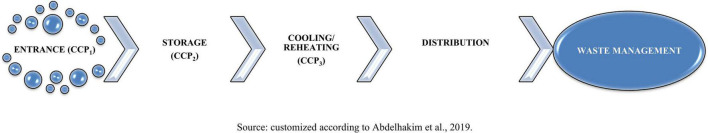
Food handling during flights and related critical control points (CCP). Source: Customized according to Abdelhakim et al. (2019).

The quality of food drinks and beverages served on long-distance flights must be at the highest level. There are absolutely no exceptions to the quality of food served in relation to the circumstances before the COVID-19 pandemic. Therefore, staff must be educated in their knowledge of the basic segments of the Hazard Analysis Critical Control Point (HACCP) food safety system. The cabin crew handles risky foods on the flight, for example, fresh salads, meat and fish dishes, or beverages with milk. The quality of food drinks and beverages served on airplanes is significantly affected by the supply chain (Sundarakani et al., 2018). If food is not loaded on the plane with care and under rigorous criteria, microbiological, chemical, physical, or allergic risks can occur. Poor handling of food by the cabin crew resulted in eight of the twelve reported cases of food poisoning due to abuse and unhygienic behavior. Examples of such reported abuses include the consumption of a meal that the passenger brought in on the flight (Abdelhakim et al., 2019). In the context of this topic, Figure 1 illustrates the general main steps [including critical control point (CCP)] during the handling of food, drinks, and beverages by the airline. These sequential steps are the same for different types of onboard food services.

Determining the quality of food, drinks, and beverages and services on the plane during the flight is one of the most important activities when planning meals for passengers on long-distance flights. This implies parameters such as: the time interval of service provision and meal consumption, different needs of passengers (changed diets, cultural habits, etc.), the capacity of the kitchen on the plane, season, and price-quality ratio of food, drinks, and beverages provided by catering (Jones, 2004; Gunardi et al., 2018; Fu, 2019; Han et al., 2020). The quality of food and beverage service is of great importance for attracting and retaining loyal customers (Chang and Yeh, 2002; Gursoy et al., 2005; Liou and Tzeng, 2007). It is extremely important for airlines not only to understand passengers’ perceptions of their service offerings but also to find out what customers expect and what types of services customers consider most important (Chen and Chang, 2005). Most of the challenges related to the provision of food, drinks, and beverage services during the flight are related to safety and quality, while for determining the amount of food, the most common guidelines are the amounts of food waste that is not consumed during the flight. These include safe food delivery, safe storage, finishing, and serving food during the flight, planning balanced meals, adhering to standards and procedures in the operational process, and special training for employees regarding food safety and food waste management (Thamagasorn and Pharino, 2019). A useful technique for reducing food waste can be the implementation of various educational programs about the importance of food waste management from sociological, economic, and environmental aspects (Blešić et al., 2021b). The optimization of meals on the plane is viewed from two aspects. The first aspect is related to meeting the needs of passengers and on the other hand, food waste that is not consumed must be reduced as much as possible (Blanca-Alcubilla et al., 2018). Many airlines use outsourcing when it comes to catering. Given the aforementioned amounts of waste generated on unused food on flights, preordering meals when buying a ticket can significantly improve and facilitate the process of food optimization in the aviation industry, why do many companies rely on outsourcing food and beverage options. Outsourcing in the production of food that will be served on flights is one way to reduce costs, more efficient meal forecasting, and more stable meal quality. The main reasons for hiring external agencies include savings related to meal production costs, focus on basic work technology (in this case, attention to improving the provision of adequate transportation services), and maintaining management flexibility (Megodawickrama, 2018).

As a result of numerous omissions, the aviation industry has recorded several foodborne epidemics. Incidents have provided an opportunity to learn from past mistakes and the current practice is to introduce high safety standards and procedures to minimize the risk of food poisoning (McMullan et al., 2007). Quality and safe service of food, drinks, and beverages on medium- and long-distance flights rely on high standards of food preparation and storage, which applies to airport kitchens, onboard service stations, and food distribution vehicles. This is particularly challenging in certain countries that do not have developed food safety management systems or food distribution conditions are difficult due to climate and social conditions. Diseases and food poisoning on planes can be very dangerous. To ensure that foodborne illness does not incapacitate the entire flight crew, crew members should consume different meals prepared by different chefs (Eisenberg et al., 1975). Food preparation for the aviation industry is a particularly sensitive process. When talking about the application of the HACCP principle related to a certain topic, it is necessary to point out that this segment is mostly guided by certain examples, based on the guidelines of the International Flight Services Association (IFSA, 2016). The IFSA recommendations recognize the following hazardous raw materials used in food production: food recalled by the local regulatory authority or food involved in the investigation of foodborne illness; raw or undercooked food of animal origin; fresh or undercooked food of plant origin; toxic substances; locally identified potentially unsafe foods (e.g., repeated unacceptable microbiological findings or government warnings); and food ingredients that may be harmful to certain consumer segments like allergens. For people who have food allergies, consuming food outside the home carries more health risks, which requires detailed control of ingredients added to meals and dressings, and control of foods that may be cross-contaminated with allergens during the food preparation process (Ahuja and Sicherer, 2007; Aleksić et al., 2020). An entity engaged in the provision of food and beverage services whose management is focused on standardized and sustainable quality must pay attention to effective communication, which is the key to risk management of food allergens (Aleksić et al., 2020). Due to the high risk of allergic reactions, Seidenberg et al. (2020) state that it is necessary to provide information on the type and severity of allergies in time, before booking a flight. Therefore, according to a study by Popov-Raljić et al. (2017), one of the primary tasks of management is to provide appropriate education to raise employee awareness of the risks that can be caused by allergenic ingredients in food and beverages.

The influence of external and internal factors on the behavior of modern consumers plays a key role in identifying needs and meeting them to achieve the goal of the sales market (Gajić et al., 2022). A recent study on large samples of respondents increases the importance and adds value to the results obtained (Durana et al., 2021a; Kovacova and Lewis, 2021; Valaskova et al., 2021a,b). Many theories such as the analytic hierarchy process (AHP) or the Dickinson model are often applied in solving complex problems that consist of numerous elements, which contain aims, criteria, subcriteria, and alternatives (Blešić et al., 2021a; Durana et al., 2021b). The Theory of Planned Behavior (TPB) is one of the most important theories used in studying consumer behavior, and predicting their future behavior and has had some success in explaining travel behavior choices, especially in explaining willingness to reduce car use (Bamberg and Schmidt, 2003; Abrahamse et al., 2005), followed by increased use of public transport (Heath and Gifford, 2002) and proenvironmental behaviors (Davison et al., 2014). However, researchers have also identified that individuals do not always act in their own rational self-interest and that a mixture of self-interest and prosocial motives may provide a better explanation for an individual’s behavior (Bamberg and Möser, 2007).

Perspectives on the recovery processes of the Serbian aviation industry are based on two sides: passenger demand and supply. In the transition to the normalization process, airlines need to review their food and beverage service strategies to increase passenger traffic, reduce competitiveness, and undermine passenger refight intentions. Uncertainty in the behavior of passengers during the normalization process causes uncertainty regarding the future projections of the aviation industry. In this context, this study aims to uncover the predictors that influenced the airline’s passenger intent to travel in the COVID-19 era, based on the Theory of Planned Behavior (TPB). The TPB explains the behavior with predictors that affect behavioral intention and states that the main driving force of the behavior is the intention to realize the behavior. Attitudes, subjective norms, intentions, and behaviors of Serbian passengers and employees on medium- and long-distance flights were examined to determine whether the quality of food, drinks, and beverages services during the flight can influence intentions that lead to airline reselection behavior through the Ajzen’s Theory of Planned Behavior (TBP) (Ajzen, 1985, 1991).

Attitudes are expressed behavior-oriented positive or negative approaches (Ajzen, 1985) and subjective norms as opinions are a stronger predictor of intention to engage in physical activity among those who may be more sensitive to others’ opinion (Latimer and Ginis, 2005). Therefore, the first and second hypotheses of this study are:

Hypothesis 1 (H1): Attitudes about food, drinks, and beverages quality and safety will significantly affect reflight intentions.
Hypothesis 2 (H2): Subjective norms will significantly affect reflight intentions.

According to a study by Ajzen (1991), perceived behavioral control is an additional determinant of intention and behavior and is defined as the composition of control beliefs of an individual about how easy or difficult it will be to perform a behavior. Therefore, the third hypothesis of this study is:

Hypothesis 3 (H3): Perceived behavioral control (PBC) will significantly affect reflight intentions.

In accordance with the aim of this study and to determine the importance and significance of causal relationships between groups of variables, three more following hypotheses were made:

Hypothesis 4 (H4): Intention mediates the relationship between attitudes and reflight behavior.
Hypothesis 5 (H5): Intention mediates the relationship between subjective norms and reflight behavior.
Hypothesis 6 (H6): Intention mediates the relationship between perceived behavioral control (PBC) and reflight behavior.

## Materials and Methods

### Data Collection and Analysis

Two preliminary questionnaires were adjusted according to a study by Polat et al. (2021) and a pilot study was conducted according to a sample of 20 voluntary respondents to determine whether the questions were clear, understandable, and suitable for further statistical processing of the obtained data, after which some of the questions were reformulated and removed.

This study was conducted with two questionnaires from November 20, 2020 to January 15, 2021. The sample consisted of 316 passengers and 55 cabin crew representatives. In final processing, the answers received from 315 passengers and from 54 employees on medium- and long-duration flight routes were used. Two questionnaires designed by the authors were used for the purposes of the survey: The first questionnaire was intended for air passengers and the second questionnaire was intended for cabin crew. In the first part, respondents evaluated on the Likert scale ranged from 1 (strongly disagree) to 7 (strongly agree), their perception in terms of attitudes about food and beverage (13 questions), subjective norms (5 questions), perceived degree of control (4 questions), intentions related to the influence of food and beverages quality and safety on air flights on reflight choice (5 questions), and behavior (6 questions). For each of the mentioned aspects, the passengers were asked a series of questions through which the observed aspects were comprehensively considered. The second part of the questionnaire collected data on the demographic characteristics.

The measurement variables and measurement items used in this study are shown in Table 1.

**TABLE 1:** Survey questionnaire and sources.

Costruct	Item (customized according to source)	References
Attitudes	The choice of food on plane is an important factor of passenger’s satisfaction Food safety is an important factor of passenger’s satisfaction on the plane The amount of food is an important factor of passenger’s satisfaction The appearance of the meal served is an important factor of passenger’s satisfaction The taste of the food is an important factor of passenger’s satisfaction The variety of food is an important factor of passenger’s satisfaction The frequency of service is an important factor of passenger’s satisfaction on the plane The freshness of the food is an important factor of passenger’s satisfaction on the plane Quality food supply is an important factor of passenger’s satisfaction on the plane Meal choice for passengers with specific diets (vegetarian, gluten-free, halal, kosher, etc.) is an important factor of passenger’s satisfaction The offer of local dishes is an important factor of passenger’s satisfaction on the plane Wide choice of drinks is an important factor of passenger’s satisfaction Wide choice of good quality drinks is an important factor of passenger’s satisfaction	Cronin et al., 2000; Abbas and El Gamal, 2015; Río et al., 2019; Duda-Chodak et al., 2020
Subjective norms	My environment expects me to use flights with a good offer of food My family expects me to use flights with a good offer of food Friends expect me to use flights with a good offer of food My business partners expect me to use flights with a good offer of food Traveling on flights with a good offer of food is a measure of social status	Latimer and Ginis, 2005; Lien et al., 2019
Perceived behavioral control	Flight dates with a good food offer do not suit my needs Prices of flights with a good food offer are too high for me Flights with a good food offer are overbooked Information on food offerings on flights is not available	Polat et al., 2021
Intentions	I will pay attention to the food offer on the flight when choosing a flight I will set aside more money for a flight that I know includes good food I will avoid flights that do not offer any food I will avoid flights on which the food offer is not good I will avoid flights on which food supply is poor	Abbas and El Gamal, 2015; Mazura et al., 2020
Behavior	I choose flights on which there is a good supply of food I choose flights on which the food offer is diverse I choose flights that offer quality food I choose the flights with the lowest price I choose the flights with the shortest duration I choose the flights with the best price-quality ratio including food	Polat et al., 2021

The calculation of the reliability coefficient is performed based on knowledge of the matrix of variance and covariance. The Cronbach’s alpha value was measured for all the predictors in the SPSS Analysis of Moment Structures (AMOS) software, version 26.00. Path analysis was performed in the SPSS AMOS software, version 26.00.

### Measurement Model

The path analysis method was designed by Wold (1974, 1985) for the analysis of high-dimensional data in a low-structure environment. Path models are defined by two sets of linear equations. First is the inner model that specifies the relationships between unobserved or latent variables, while the other model specifies the relationships between a latent variable and its observed or manifest variables. The path model is based on the least squares estimate with the primary goal of maximizing the explanation of the variance in the construction that depends on the model of the structural equation (Henseler et al., 2009).

**FIGURE 2:**
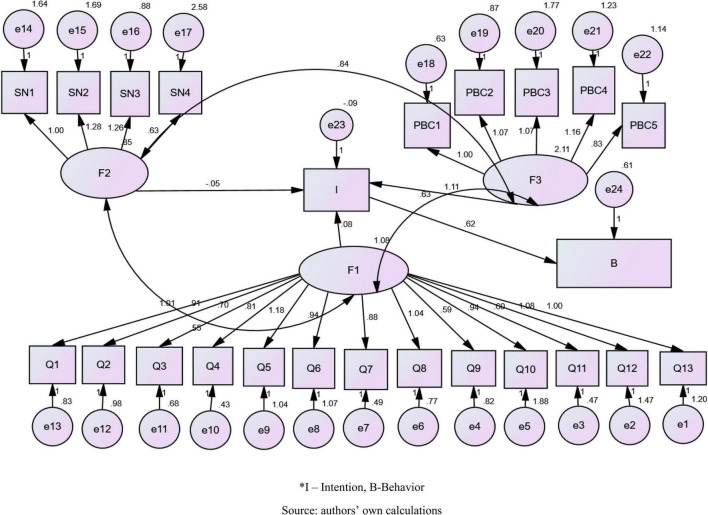
Standardized path coefficients. *I, intention; B, behavior. Source: authors’ own calculations.

The hypotheses were tested through path analysis in the AMOS using the endogenous variable intention (Abbas and El Gamal, 2015; Mazura et al., 2020) regressed on the variables attitude (Cronin et al., 2000; Abbas and El Gamal, 2015; Río et al., 2019; Duda-Chodak et al., 2020), subjective norms (Latimer and Ginis, 2005; Lien et al., 2019), and perceived behavioral control (Polat et al., 2021). The standardized path coefficients generated in the AMOS path analysis are shown in Figure 2.

## Results

### Study Sample

Most of the respondents (86%) belonged to the working population and mostly ranged in age from 25 to 45 years. Among the passengers and employees who took part in the survey, women (65%) were more represented than men. Respondents from the cabin crew were employed by Air Serbia and were mostly engaged in long- and medium-duration flights where complex in-flight food, drinks, and beverages offerings are common.

### Results of Path Analyses

The aim was to examine the importance and significance of causal relationships between the groups of variables. The calculation of the reliability coefficient is performed based on knowledge of the matrix of variance and covariance. According to some divisions, the measurement is reliable considering the value of the coefficient α (Cronbach’s alpha). The Cronbach’s alpha value was measured for all the predictors with the following values: Quality (α = 0.921) (13 items), attitudes (α = 0.878) (five items), subjective norms (α = 0.691) (four items), PBC (α = 0.909) (five items), intentions (α = 0.664) (six items), and behavior (α = 0.711) (four items).

Regression analysis concluded that certain predictors such as food service quality and safety significantly affect the attitude, subjective norms, and perceived behavioral control (PBC) of passengers and trigger the intention that affects behaviors in the choice of the airline during the COVID-19 pandemic, especially when it comes to flights with medium and long durations. The hypotheses were tested through path analysis in the AMOS using the endogenous variable intention regressed on the variables attitude, subjective norms, and perceived behavioral control. The standardized path coefficients generated in the AMOS path analysis are shown in Figure 2.

The path diagram (Figure 2) provides an insight into causal relationships. Arrows from one observed variable to another observed variable indicate the functional relationships between variables. Factor load (correlation coefficient between each variable, i.e., the question and the factor itself) is shown on the arrows. Questions of similar content can be linked to get a better fit of the model. In the given chart, for the sake of clarity of work, there will be no connection between errors.

Parameters showing the absolute fit indices of the model rely heavily on conventional limit values that are as follows: chi-square = 2161.218, *df* = 247, *p* = 0.00, Root Mean Square Error of Approximation (RMSEA) = 0.63, Comparative Fit Index (CFI) = 0.989 and Tucker-Lewis index (TLI) = 0.960, chi-square value (CMIN) = 3.753, Akaike information criterion (AIC) = 2267.218, and Bayesian information criterion (BIC) = 2474.490.

This model achieves compatibility with all the collected data. After the model has been fitted, parameter estimates are displayed in the table. Parameter estimates for the means structure model in Table 1 show the statistical significance of the causal relationships of the variables, which indicates the fact that intention is a significant mediator between the variables of quality, perceived behavioral control (PBC), and behavior.

It is noticed that factor F2 (social norms) does not make a partial contribution to the influence on the variable intentions. The perception of the risk of the COVID-19 virus is individual, so family, immediate environment, and social status did not have a decisive influence on the intentions and behaviors related to the consumption of food and beverages during the flight.

Factor F1 (quality) significantly contributes to the impact on intentions with values: *p* = 0.03 and estimates = 0.80. Attitudes about the quality and safety of food, drinks, and beverages served during the flight had a significant influence on intentions because of the high level of risk during the consumption and fear of cause in unusual circumstances of the COVID-19 pandemic. Also, factor 3 (perceived behavioral control) shows a correlation with intentions (*p* = 0.00, estimates = 1.11) because of the availability and timed information about the quality and safety of food and beverage services during medium- and long-distance flights.

Results shown in Table 2 indicate a significant influence of intentions on behavior (*p* = 0.00, estimates = 0.618).

**TABLE 2:** Parameter estimates for means structure model.

			Estimate	S.E.	C.R.	*P*	Label
I	<- -	F2	–0.053	0.043	–1.245	0.213	
I	<- -	F1	0.080	0.027	2.994	0.003	
I	<- -	F3	1.114	0.035	31.959	***	
Q13	<- -	F1	1.000				
Q12	<- -	F1	1.078	0.088	12.203	***	
Q11	<- -	F1	0.605	0.050	12.138	***	
Q9	<- -	F1	0.586	0.058	10.144	***	
Q10	<- -	F1	0.942	0.089	10.540	***	
Q8	<- -	F1	1.043	0.075	13.865	***	
Q7	<- -	F1	0.885	0.063	14.154	***	
Q6	<- -	F1	0.941	0.076	12.334	***	
Q5	<- -	F1	1.180	0.086	13.718	***	
Q4	<- -	F1	0.806	0.057	14.029	***	
Q3	<- -	F1	0.698	0.059	11.907	***	
Q2	<- -	F1	0.909	0.073	12.407	***	
Q1	<- -	F1	1.010	0.075	13.504	***	
SN1	<- -	F2	1.000				
SN2	<- -	F2	1.284	0.139	9.263	***	
SN3	<- -	F2	1.263	0.128	9.876	***	
SN4	<- -	F2	0.634	0.115	5.508	***	
PBC1	<- -	F3	1.000				
PBC2	<- -	F3	1.066	0.044	24.115	***	
PBC3	<- -	F3	1.072	0.055	19.361	***	
PBC4	<- -	F3	1.155	0.051	22.859	***	
PBC5	<- -	F3	0.826	0.044	18.790	***	
B	<- -	I	0.618	0.026	24.132	***	

I, intentions; Q, questions; SN, subjective norms; PBC, perceived behavioral control; B, behavior; ***Statistical significance.

*Source: Authors’ own calculations.*

Table 3 shows good and positive correlations between all three factors. It can be noticed that factors F2 and F3 share 37.4% of the variance, F1 and F2 share 32.4% of the variance, and F1 and F3 share only 16.8% of the variance.

**TABLE 3:** Correlations between factors.

			Estimate	%
F2	<- ->	F3	0.628	38.4%
F1	<- ->	F2	0.576	32.4%
F1	<- ->	F3	0.419	16.8%

*Source: Authors’ own calculations.*

## Discussion

The COVID-19 pandemic changed consumer eating habits (Polat et al., 2021; Bakır et al., 2022) and conditioned airlines to focus on improving the overall quality and safety of food drinks and beverages during medium- and long-distance flights. Consumer concerns about food safety have increased during the COVID-19 pandemic period, but there is no evidence that viruses that cause respiratory diseases are transmitted through food. This finding may be a direct consequence of the fact that food contact is not considered completely safe in the context of the COVID-19 pandemic (Duda-Chodak et al., 2020). Before the COVID-19 pandemic, passenger satisfaction with meals on the plane had a significant impact on their loyalty and intention to fly again and recommend the used airline services to others (Abbas and El Gamal, 2015). Perceived quality could have a positive impact on satisfaction, while satisfaction could have a positive impact on the image, intentions, and behavior of passengers related to the choice of the airline (Maeng and Park, 2015). Uncertainty in passenger behavior during the normalization process causes uncertainty about the future projections of the aviation industry, especially when it comes to high-risk services such as the consumption of food drinks and beverages during flights (Polat et al., 2021).

Previous authors (Cronin et al., 2000; Abbas and El Gamal, 2015) state that in the service industry, service quality and consumer satisfaction have been linked together. Previous experiences and attitudes of passengers related to air transport are approximately the same. Practices created in emergency situations, such as the COVID-19 pandemic, could lead to a significant change in attitudes related to the quality of food and beverage service in airplanes. Many airlines have improved the safety procedures and quality of food drinks and beverages, especially from the point of view of retaining satisfied passengers. People’s attitude toward behavior, subjective norm, and perceived control of behavior initiates the intention of behavior (Ajzen, 1991). Results showed that attitudes about food and beverages service quality have a direct influence on intention and an indirect effect on customer behavior, which confirms hypothesis 1 (H1).

Subjective norms or beliefs about whether social circles that influence an individual’s behavior will approve of that behavior emphasize the perception of social pressure to perform a certain action, which classifies them as a function of belief (Ajzen, 1991; Lien et al., 2019). So, references that an individual feels close to greatly influence the personal decision or behavior (Han et al., 2011). Traveling on flights with quality food can be a reflection of social status. The opinions of business partners, family, or expectations of the immediate environment can influence the decision to choose airlines that provides quality and safe food, drinks, and beverage services during the flight. When it comes to the choice of airlines in normal conditions, subjective norms can easily affect intentions for subsequent behaviors, but in the case of extreme conditions, the results showed the opposite, which denied hypothesis 2 (H2).

In the context of the quality of food, drinks, and beverage services provided onboard, the service environment also plays a significant role. The passenger can be essentially satisfied with the food presented, but he does not have to like the service environment. Messner (2016) states that the perception of food quality is primarily influenced by the quality of cabin crew service and the specific environment. Perceived risk is defined as a consumer’s perception of the possibility of unknown bad consequences from past experiences and it is influenced by individual psychological and environmental factors (Kim et al., 2008; Cho et al., 2018). According to a study by Ajzen (1991), perceived behavioral control can be used to predict the probability of a successful behavioral intention. Results indicated that perceived behavioral control significantly affects reflight intentions, which confirms hypothesis 3 (H3).

Cretan behaviors can be predicted from intentions with considerable accuracy. An individual’s past experiences may affect his or her intentions and future behavioral performance (Ajzen, 1991; Ajzen and Fishbein, 2005). Good examples can be found in behaviors that involve a choice among available alternatives. According to the results, the intention is the key mediator between independent variables and attitudes about food quality and safety, subjective norms, perceived behavior control, and reflight behavior that confirm hypothesis 4 (H4), hypothesis 5 (H5), and hypothesis 6 (H6).

## Conclusion and Limitations

The main objective of this study was to improve the quality of food and beverages served in flight according to the circumstances in extreme conditions of COVID-19. Inflight food, drinks, and beverage services are a key element of the air transport value chain and to accommodate demands in a safe and efficient way. It can be concluded that a better understanding of passengers’ intentions can make the airlines more sensitive and effective in operation management techniques and improve passengers’ satisfaction and, also, gain reflight behavior. Table 4 shows the following acceptability of the hypothesis.

**TABLE 4:** Acceptability of the hypothesis.

Hypothesis	Hypothesis description	Acceptability of the hypothesis
Hypothesis (H_1_)	Attitudes about food, drinks and beverages quality and safety will significantly affect re-flight intentions	Confirmed
Hypothesis (H_2_)	Subjective norms will significantly affect to re-flight intentions	Denied
Hypothesis (H_3_)	Perceived Behavioral Control (PBC) will significantly affect re-flight intentions	Confirmed
Hypothesis (H_4_)	Intention mediates the relationship between attitudes and re-flight behavior	Confirmed
Hypothesis (H_5_)	Intention mediates the relationship between subjective norms and re-flight behavior	Confirmed
Hypothesis (H_6_)	Intention mediates the relationship between perceived behavioral control (PBC) and re-flight behavior	Confirmed

*Source: Author’s research.*

This study provides new results related to consumers’ reflight subsequent behavioral intentions in unknown conditions and indicates that they are not influenced by social norms but by individual perceptions of food and beverage quality and safety and perceived behavioral control. The significance of this study is in the qualification of factors that influence the intention of future behavior and shows how attitudes about the quality and safety of food and beverages consumed during the flight can influence the reselection of the airline in uncertain conditions caused by the COVID-19 pandemic. Building on global best practices regarding COVID-19, it is necessary to create protocols related to the provision of food and beverage services, which would prevent unforeseen risks through continuous implementation.

## Further Study

Recommendation for future study should be related to the recovery period where some, seemingly neglecting, factors may be crucial to differentiate from competing airlines and to introduce more factors that make up the extended model of the Theory of Planned Behavior. A larger number of respondents would provide more guidance on potential opportunities to improve and refine the operational processes of providing food and beverage services during the flight.

## Data Availability Statement

The raw data supporting the conclusions of this article will be made available by the authors, without undue reservation.

## Ethics Statement

Ethical review and approval was not required for the study on human participants in accordance with the local legislation and institutional requirements. The patients/participants provided their written informed consent to participate in this study.

## Author Contributions

MA, JP, and TG contributed to study conceptualization, study design, leading statistical analyses and interpretation of results, and writing the original text. IB, MD, MP, and JB contributed to the study by providing comments to refine the manuscript. All authors have contributed to the article and approved the submitted version of the manuscript.

## Author Disclaimer

All the claims expressed in this article are solely those of the authors and do not necessarily represent those of their affiliated organizations or those of the publisher, the editors, and the reviewers. Any product that may be evaluated in this article or claim that may be made by its manufacturer is not guaranteed or endorsed by the publisher.

## Conflict of Interest

The authors declare that the research was conducted in the absence of any commercial or financial relationships that could be construed as a potential conflict of interest.

## Publisher’s Note

All claims expressed in this article are solely those of the authors and do not necessarily represent those of their affiliated organizations, or those of the publisher, the editors and the reviewers. Any product that may be evaluated in this article, or claim that may be made by its manufacturer, is not guaranteed or endorsed by the publisher.
